# Telestroke and Timely Treatment and Outcomes in Patients With Acute Ischemic Stroke

**DOI:** 10.1001/jamanetworkopen.2025.34275

**Published:** 2025-09-26

**Authors:** Brian Stamm, Rachael T. Whitney, Regina Royan, Ghada Ibrahim, Adrienne V. Nickles, Rebecca A. Ferber, Wen Ye, Wan-Ling Hsu, Nikita Chhabra, Rodney A. Hayward, Mollie McDermott, Phillip A. Scott, Kevin N. Sheth, Mathew J. Reeves, Deborah A. Levine

**Affiliations:** 1 Department of Neurology, University of Michigan, Ann Arbor; 2 Institute for Healthcare Policy and Innovation, University of Michigan, Ann Arbor; 3 VA Center for Clinical Management Research, VA Ann Arbor Healthcare System, Ann Arbor, Michigan; 4 Geriatric Research Education and Clinical Center, VA Ann Arbor Healthcare System, Ann Arbor, Michigan; 5 Department of Internal Medicine, University of Michigan, Ann Arbor; 6 Department of Emergency Medicine, University of Michigan, Ann Arbor; 7 Assistant Editor, *JAMA Network Open*; 8 Michigan Department of Health and Human Services, Lansing; 9 Department of Biostatistics, University of Michigan School of Public Health, Ann Arbor; 10 Center for Brain and Mind Health, Departments of Neurology and Neurosurgery, Yale School of Medicine, Yale University, New Haven, Connecticut; 11 Department of Epidemiology and Biostatistics, Michigan State University, East Lansing

## Abstract

**Importance:**

Telestroke has the potential to revolutionize acute stroke treatment by improving access to optimal stroke care, including time-sensitive care such as thrombolysis. However, it is unclear how treatment times and stroke outcomes compare between patients evaluated and not evaluated by telestroke.

**Objective:**

To evaluate the association between telestroke use and acute stroke treatment times and outcomes.

**Design, Setting, and Participants:**

This retrospective cohort study included patients with acute ischemic stroke aged 18 years or older presenting to 42 Paul Coverdell Michigan Stroke Registry hospitals from January 1, 2022, to December 31, 2023. All patients were potentially eligible for thrombolysis (ie, presented ≤4 hours of last known well, no contraindications to thrombolysis documented).

**Exposure:**

Telestroke (vs nontelestroke) encounter.

**Main Outcomes and Measures:**

The primary outcomes were receipt of thrombolysis and door-to-needle (DTN) time as a continuous variable and a categorical variable (≤60 vs >60 minutes). Secondary outcomes included discharge ambulatory status, discharge destination, and door-in-door-out (DIDO) time in transferred patients. Multivariable hierarchical models evaluated associations between telestroke (vs nontelestroke) activation and outcomes, sequentially adjusting for demographics, medical history, presentation or arrival, and hospital characteristics.

**Results:**

Among the 3036 patients with acute ischemic stroke potentially eligible for thrombolysis (mean [SD] age, 69.7 [14.5] years; 1563 male [51.5%]), 785 (25.9%) were evaluated using telestroke and 2251 (74.1%) without telestroke. A total of 1673 patients (55.1%) were treated with thrombolysis. In the fully adjusted models, patients evaluated by telestroke had a significantly higher odds of receiving thrombolysis (adjusted odds ratio, 1.61; 95% CI, 1.17-2.23) but longer DTN times (6.55 minutes longer; 95% CI, 2.12-10.97 minutes longer) and lower odds of meeting a guideline-concordant DTN time within 60 minutes (adjusted odds ratio, 0.56; 95% CI, 0.39-0.81) compared with those not evaluated by telestroke. Among 255 patients who underwent interhospital transfer, 207 (81.2%) received thrombolysis, and patients with telestroke had significantly longer DIDO times (46.90 minutes longer, 95% CI, 1.08-92.72 minutes longer).

**Conclusions and Relevance:**

In this cohort study of patients with acute ischemic stroke potentially eligible for thrombolysis, those evaluated by telestroke had a 61% higher odds of receiving thrombolysis but a 44% lower odds of meeting guideline-concordant DTN times within 60 minutes and prolonged DIDO times compared with those not evaluated by telestroke. Future research should investigate modifiable factors that contribute to treatment delays in patients with ischemic stroke evaluated via telestroke.

## Key Points

**Question:**

How do treatment times and stroke outcomes in patients with acute ischemic stroke evaluated by telestroke compare with those not evaluated by telestroke?

**Findings:**

In this cohort study of 3036 patients with acute ischemic stroke potentially eligible for thrombolysis, telestroke was associated with a higher odds of receiving thrombolysis, but significantly prolonged door-to-needle and door-in-door-out times and a lower odds of meeting guideline-concordant door-to-needle times within 60 minutes, compared with nontelestroke.

**Meaning:**

These findings suggest that there is room to improve timely stroke treatment for patients evaluated by telestroke to ensure that all patients with ischemic stroke receive guideline-concordant, time-sensitive care.

## Teaser

This cohort study evaluates the association of telestroke use with time to treatment and outcomes among patients with acute ischemic stroke in Michigan.

## Introduction

Acute ischemic stroke (AIS) treatment is not only highly efficacious but also highly time-sensitive.^1,2^ Faster time to treatment with intravenous thrombolysis (door to needle [DTN]) is associated with improved outcomes in AIS.^1^ For patients with a large vessel occlusion, approximately 1.9 million neurons die during each minute without treatment.^3^ Target: Stroke is a national quality improvement campaign by the American Heart Association (AHA) that achieved significant overall reductions in DTN times.^4,5,6^ Since its inception in 2010, Target: Stroke has set increasingly bold benchmarks for DTN times, with the most recent goal of achieving a DTN within 60 minutes in 85% of patients treated with intravenous thrombolysis.^7^

While thrombolysis is highly efficacious for acute stroke, not all hospitals have stroke specialists. Telestroke has revolutionized the treatment of acute stroke by improving access to expert stroke care.^8^ Telestroke connects stroke specialists (typically from a comprehensive stroke center or hub site) to spoke sites (typically primary stroke centers) to aid in acute stroke decision-making and treatment.^9^ Telestroke has led to an increased uptake of acute stroke therapies, including thrombolysis,^8^ and has grown across the nation, becoming a vital component of stroke systems of care.^8,10^ However, several studies from single systems found that telestroke treatment times at spoke hospitals, including DTN times, were significantly prolonged,^11,12,13,14,15^ with worse inpatient and discharge stroke outcomes^12^ compared with standard, in-person stroke care.

A larger-scale comparison of telestroke vs standard stroke care has been difficult to perform given challenges with identifying patient-level telestroke encounters in standard administrative data. The Paul Coverdell Michigan Stroke Registry started routinely tracking telestroke encounters in 2022. This study used this state-level stroke registry to compare thrombolysis treatment rates, DTN times, and other evidence-based stroke hospitalization and discharge outcomes in patients evaluated via telestroke vs not.

## Methods

In this cohort study, data were obtained from the Paul Coverdell Michigan Stroke Registry, a Centers for Disease Control and Prevention–funded, state-level program that collects stroke care data from the AHA’s Get With The Guidelines–Stroke registry to measure, track, and improve the quality of care for patients with stroke. Michigan has participated in the Coverdell program since its inception in 2003.^16^ The institutional review boards at both the Michigan Department of Health and Human Services and the University of Michigan approved this study. As the study was a secondary analysis of deidentified data, the institutional review boards exempted the study from informed consent. This study follows the Strengthening the Reporting of Observational Studies in Epidemiology (STROBE) reporting guideline.^17^

### Study Population

All patients aged 18 years or older presenting to Paul Coverdell Michigan hospitals between January 1, 2022 (when the telestroke variable was first routinely used), and December 31, 2023, with a diagnosis of AIS and a nonmissing response to the Get With The Guidelines–Stroke question, “Was telestroke consultation performed,” were included. Patients with any of the following criteria were excluded: (1) diagnosis of intracerebral hemorrhage, subarachnoid hemorrhage, or transient ischemic attack; (2) inpatient stroke; (3) transported by mobile stroke unit; (4) ineligible for thrombolysis (last known well–to–emergency department arrival time >4 hours or met other standard exclusion criteria)^18^; and (5) missing data on hospital arrival method, receipt of thrombolysis, National Institutes of Health Stroke Scale (NIHSS) score at admission, or other key covariates as detailed herein the Methods. For patients who underwent an interhospital transfer, data were obtained from the sending facility. eFigure 1 in Supplement 2 depicts the study derivation.

### Primary Exposure

The primary independent variable was telestroke activation (vs no telestroke activation). Telestroke activation was defined by a response of, “Yes, the patient received telestroke consultation from a remotely located expert when the patient was located at my hospital,” to the to the Get With The Guidelines–Stroke question, “Was telestroke consultation performed?”

### Outcomes

The primary outcomes were receipt of thrombolysis and DTN time as continuous and categorical variables (≤60 vs >60 minutes) per current AIS guidelines.^19^ Secondary outcomes included postthrombolytic symptomatic intracerebral hemorrhage, in-hospital mortality, ordinal discharge modified Rankin Scale (mRS) score, discharge ambulatory status (ambulatory vs nonambulatory), discharge destination (home vs other [acute care facility, other health care facility, left against medical advice, hospice, or death]), and DTN time (≤45 vs >45 minutes), per Target: Stroke recommendations,^7^ and among those transferred to another hospital, door-in-door-out (DIDO) time (minutes).

### Prespecified Covariates

Patient-level demographics included age (years), sex (male vs female), race and ethnicity (Hispanic, non-Hispanic Black [hereafter, Black], non-Hispanic White [hereafter, White], non-Hispanic other [Asian, American Indian or Alaska Native, Native Hawaiian or Pacific Islander, and unable to determine; hereafter, other] as abstracted from the medical record). Medical history variables included atrial fibrillation, stroke, heart failure, diabetes, hypertension, and cigarette smoking. Race and ethnicity were included as covariates given known racial and ethnic disparities in various acute stroke processes.

Presenting and arrival covariates included last known well–to–arrival time (per minute increase), large vessel occlusion, NIHSS score categories (0-4 [no or minor stroke], 5-15 [moderate stroke], 16-20 [moderate to severe stroke], 21-42 [severe stroke]),^20^ arrival method (eg, use of emergency medical services [EMS] vs private transportation), EMS prenotification (advanced emergency department notification by EMS of suspected stroke),^21^ off-hours arrival time (regular [nonholiday] hours 7:00 am to 6:00 pm, Monday through Friday),^22^ door–to–computed tomography imaging time (≤20 minutes),^19^ and transfer to another hospital. Hospital characteristics included geographic location (metropolitan hospital, yes or no), teaching hospital (yes or no), presence of a neurology residency program (yes or no), Joint Commission stroke center status (primary stroke center, thrombectomy-capable stroke center, comprehensive stroke center, noncertified hospital),^23^ and annual number of ischemic stroke discharges (<200, 200-600, >600).

### Statistical Analysis

Descriptive statistics, including proportions, means and SDs, and medians and IQRs, were performed as appropriate. The differences in covariates, primary and secondary outcomes between the groups (telestroke vs nontelestroke) were tested using χ^2^ tests (or Fisher exact tests) and Wilcoxon rank sum tests, as appropriate. Hospital characteristics were also compared between telestroke hospitals (defined as hospitals that had at least 1 telestroke encounter during the study period) vs nontelestroke hospitals.

#### Primary Analysis

Sequential multilevel modeling was used to estimate the variation and factors associated with outcomes at the patient and hospital level. Model 1 contained the hospital-specific random intercepts to account for patient clustering within each hospital. This model was used to estimate the intraclass correlation coefficient to calculate hospital-level variation in outcomes. Model 2 added patient-level factors (including demographics, medical history, and presenting or arrival factors) to model 1. Model 3 added hospital-level covariates to model 2. These sequential models were performed for each primary outcome and for secondary outcomes with statistically significant (*P* < .05) bivariate comparisons. An exploratory model 4 tested for effect modification by adding telestroke–by–off-hours arrival time and telestroke–by–stroke center status interaction terms, respectively, into the fully adjusted DTN outcome models to examine whether delays were concentrated in specific settings.

Secondary outcome multivariable regression models were performed among patients who received thrombolysis, given the association between thrombolysis and hospital discharge outcomes.^1^ Given the relatively low sample size of patients transferred to another hospital who had received thrombolysis, the multivariable model for the secondary outcome of DIDO time only adjusted for patient-level factors, but patient clustering within hospitals was accounted for by the multilevel model.

#### Sensitivity Analysis

Given prior literature showing differential characteristics and outcomes of patients with AIS by transfer status,^24^ a sensitivity analysis was performed that removed transferred patients. An additional sensitivity analysis was performed in which missing responses to the telestroke activation variable were imputed to no.

All statistical analyses were performed using StataSE, version 19 (StataCorp LLC). Statistical significance was set at a 2-sided *P* < .05.

## Results

Among the 3036 patients with confirmed AIS who were potentially eligible for thrombolysis (mean [SD] age, 69.7 [14.5] years; 1473 female [48.5%] and 1563 male [51.5%]; 490 of Black [16.1%], 63 of Hispanic [2.1%], 2354 of White [77.5%], and 129 of other [4.2%] race and ethnicity), 785 (25.9%) were evaluated using telestroke and 2251 (74.1%) without telestroke (Table 1). Patients evaluated by telestroke had significantly shorter last known well–to–arrival times (median [IQR], 65.0 [38.0-120.0] vs 75.0 [47.0-128.0] minutes; *P* < .001), a greater proportion of large vessel occlusion (26.5% vs 21.4%; *P* = .003), higher NIHSS scores (8.5% vs 5.6% for severe stroke; *P* = .001), and a greater proportion with door–to–computed tomography time within 20 minutes (71.3% vs 65.9%; *P* = .005) (Table 1). The proportion of patients evaluated by telestroke was significantly lower at metropolitan facilities (79.4% vs 90.1%), teaching hospitals (65.9% vs 87.3%), hospitals with a neurology residency program (10.6% vs 41.2%), and comprehensive stroke centers (18.1% vs 54.2%) (all *P* < .001) (Table 1).

**Table 1.** Baseline Patient (N = 3036) and Hospital Characteristics Stratified by Telestroke Activation

Characteristic	Telestroke activation, No. (%)		*P* value^a^
	Yes (n = 785)	No (n = 2251)	
**Patient demographics**			
Age at time of stroke, median (IQR) [range], y	72.0 (62.0-81.0) [18.0-101.0]	70.0 (60.0-80.0) [18.0-102.0]	.002
Sex			
Female	403 (51.3)	1070 (47.5)	.07
Male	382 (48.7)	1181 (52.5)	
Race and ethnicity, No. (%)			
Black	95 (12.1)	395 (17.5)	.001
Hispanic	11 (1.4)	52 (2.3)	
White	646 (82.3)	1708 (75.9)	
Other^b^	33 (4.2)	96 (4.3)	
Medical history			
Atrial fibrillation or flutter	124 (15.8)	295 (13.1)	.06
Prior stroke or transient ischemic attack	246 (31.3)	669 (29.7)	.40
Prior heart failure	84 (10.7)	226 (10.0)	.60
Diabetes	220 (28.0)	689 (30.6)	.17
Hypertension	613 (78.1)	1669 (74.1)	.03
Cigarette smoking	149 (19.0)	435 (19.3)	.83
Stroke presentation			
Last known well to arrival time, median (IQR), min	65.0 (38.0-120.0)	75.0 (47.0-128.0)	<.001
Large vessel occlusion	208 (26.5)	482 (21.4)	.003
NIHSS score			
0-4 (No or mild stroke)	394 (50.2)	1291 (57.4)	.001
5-15	276 (35.2)	716 (31.8)	
16-20	48 (6.1)	117 (5.2)	
21-42 (Severe stroke)	67 (8.5)	127 (5.6)	
Arrival and transfer characteristics			
Arrival mode			
Private transportation	243 (31.0)	697 (31.0)	.20
EMS no prenotification	215 (27.4)	549 (24.4)	
EMS prenotification	327 (41.7)	1005 (44.6)	
Off-hours arrival time^c^	423 (53.9)	1276 (56.7)	.17
Door-to-CT time ≤20 min	560 (71.3)	1484 (65.9)	.005
Transferred to another hospital	71 (9.0)	19 (0.8)	<.001
**Hospital characteristics**			
Metropolitan	623 (79.4)	2028 (90.1)	<.001
Teaching	517 (65.9)	1964 (87.3)	<.001
Presence of a neurology residency program	83 (10.6)	928 (41.2)	<.001
Stroke center status			
Comprehensive	142 (18.1)	1220 (54.2)	<.001
Thrombectomy capable	111 (14.1)	156 (6.9)	
Primary	517 (65.9)	875 (38.9)	
Not certified	15 (1.9)	0	
Annual No. of acute stroke discharges			
<200	115 (14.6)	11 (0.5)	<.001
200-600	546 (69.6)	1170 (52.0)	
>600	124 (15.8)	1070 (47.5)	

Abbreviations: CT, computed tomography; EMS, emergency medical services; NIHSS, National Institutes of Health Stroke Scale.

^a^ Differences between continuous variables were assessed using Mann-Whitney *U *tests, and differences between categorical variables were assessed using χ^2^ tests.

^b^ Other included Asian, American Indian or Alaska Native, Native Hawaiian or Pacific Islander, and unable to determine.

^c^ Regular (nonholiday) hours are 7:00 am to 6:00 pm, Monday through Friday.

Thrombolysis was administered to 1673 patients, including 436 of 785 potentially eligible patients (55.5%) evaluated by telestroke vs 1237 of 2251 (55.0%) not evaluated by telestroke (*P* = .78) (Table 2). Patients evaluated by telestroke had longer median DTN times (53.0 [IQR, 40.0-74.0] vs 46.0 [IQR, 33.0-64.0] minutes; *P* < .001). Among transferred patients, those evaluated by telestroke had longer median DIDO times (166.0 [IQR, 92.0-252.0] vs 150.0 [IQR, 103.0-235.5] minutes; *P* < .001). Fewer patients evaluated by telestroke met guideline-concordant DTN times within 60 minutes compared with those not treated by telestroke (263 of 436 [60.3%] vs 894 of 1237 [72.3%]; *P* < .001).

**Table 2.** Primary and Secondary Outcomes Stratified by Telestroke Activation, Unadjusted

Outcome	Patients eligible for thrombolysis (N = 3036)			Patients who received thrombolysis (n = 1673)		
	Telestroke activation (n = 785)	No telestroke (n = 2251)	*P* value^a^	Telestroke activation (n = 436)	No telestroke (n = 1237)	*P* value^a^
**Primary**						
Thrombolysis administered, No. (%)	436 (55.5)	1237 (55.0)	.78	NA	NA	NA
DTN time, median (IQR), min	NA	NA	NA	53.0 (40.0-74.0)	46.0 (33.0-64.0)	<.001
**Secondary outcomes**						
Postthrombolytic ICH, No. (%)	NA	NA	NA	11 (2.5)	33 (2.7)	.87
In-hospital mortality, No. (%)	23 (2.9)	67 (3.0)	.95	15 (3.4)	52 (4.2)	.48
Discharge mRS, median (IQR), points^b^	2.0 (0.0-4.0)	1.0 (0.0-4.0)	.008	2.0 (1.0-4.0)	2.0 (1.0-4.0)	.008
Discharge mRS, No. (%), points^b^						
0 (No symptoms)	88 (25.4)	372 (28.2)	.008	39 (20.2)	175 (22.9)	.10
1	75 (21.6)	334 (25.3)		48 (24.9)	184 (24.1)	
2	39 (11.2)	136 (10.3)		24 (12.4)	89 (11.6)	
3	27 (7.8)	137 (10.4)		11 (5.7)	80 (10.5)	
4	68 (19.6)	235 (17.8)		37 (19.2)	155 (20.3)	
5	27 (7.8)	56 (4.2)		19 (9.8)	42 (5.5)	
6 (Death)	23 (6.6)	49 (3.7)		15 (7.8)	40 (5.2)	
Ambulatory status at discharge, No. (%)^c^						
Independent	420 (62.7)	1225 (70.1)	<.001	208 (60.1)	614 (65.8)	.01
With assistance from another person	166 (24.8)	386 (22.1)		84 (24.3)	226 (24.2)	
Unable	84 (12.5)	136 (7.8)		54 (15.6)	93 (10.0)	
Discharge destination, No. (%)						
Home	441 (56.2)	1409 (62.6)	<.001	441 (56.2)	1409 (62.6)	<.001
Acute care facility	90 (11.5)	25 (1.1)		90 (11.5)	25 (1.1)	
Other health care facility	179 (22.8)	616 (27.4)		179 (22.8)	616 (27.4)	
Left against medical advice	13 (1.7)	46 (2.0)		13 (1.7)	46 (2.0)	
Hospice or died	62 (7.9)	155 (6.9)		62 (7.9)	155 (6.9)	
DIDO time, median (IQR), min^d^	166.0 (92.0-252.0)	150.0 (103.0-235.5)	<.001	142.0 (88.0-230.0)	123.0 (96.0-192.0)	<.001

Abbreviations: DIDO, door-in-door-out; DTN, door-to-needle; ICH, intracerebral hemorrhage; mRS, modified Rankin Scale; NA, not applicable.

^a^ Differences between continuous variables were assessed using Mann-Whitney *U* test, and differences between categorical variables were assessed using χ^2^ test.

^b^ Assessed in a subset of patients with documented mRS score at discharge (n = 1666).

^c^ Assessed in a subset of patients who were able to ambulate independently prior to admission (n = 2653).

^d^ Assessed in a subset of patients who were transferred to another hospital (n = 255).

Nontelestroke hospitals (n = 20) compared with telestroke hospitals (n = 22) were more often teaching hospitals (20 [100%] vs 13 [59.1%]; *P* = .001), in a metropolitan location (20 [100%] vs 15 [68.2%]; *P* = .006), and with a higher annual number of acute stroke discharges (≥200 discharges: 20 [100%] vs 15 [68.2%]; *P* = .01) (eTable 1 in Supplement 1). There was variation in telestroke use at the hospital level (eFigure 2 in Supplement 2).

Calculated intraclass correlation coefficients for the empty models with only hospital random intercepts included were 0.12 for the outcome of receipt of thrombolysis and 0.10 for both continuous and dichotomous DTN time outcomes. In the fully adjusted model 3, patients evaluated by telestroke had a higher odds of receiving thrombolysis compared with those who were not (adjusted odds ratio, 1.61; 95% CI, 1.17-2.23; *P* = .003). Other factors associated with receipt of thrombolysis are listed in Table 3. After adjusting for patient-level factors, patients evaluated by telestroke had significantly longer DTN times (7.44 minutes longer; 95% CI, 2.97-11.90 minutes longer; *P* = .001). This difference was slightly attenuated after adjustment for hospital characteristics (6.55 minutes longer; 95% CI, 2.12-10.97 minutes longer; *P* = .003) (Table 4). There was no evidence of significant telestroke–by–off-hours arrival time or telestroke–by–stroke center status interactions for DTN time. In the fully adjusted multilevel model of guideline-concordant DTN time within 60 minutes, patients evaluated by telestroke had a lower odds of receiving thrombolysis within 60 minutes compared with those who were not (adjusted odds ratio, 0.56; 95% CI, 0.39-0.81; *P* = .002) (Table 5).

**Table 3.** Multilevel Model of Receipt of Thrombolysis by Telestroke Activation, With and Without Adjustment for Patient and Hospital Characteristics (N = 3036)

Coefficient	Model 1: unadjusted		Model 2: adjusted for patient characteristics^a^		Model 3: further adjusted for patient and hospital characteristics^b^	
	OR (95% CI)	*P* value	AOR (95% CI)	*P* value	AOR (95% CI)	*P* value
Telestroke activation, yes vs no	2.24 (1.70-2.94)	<.001	1.48 (1.08-2.04)	.02	1.61 (1.17-2.23)	.003
Age, per 1-y increase	NA	NA	0.99 (0.98-0.99)	<.001	0.99 (0.98-0.99)	<.001
Sex, female vs male	NA	NA	0.84 (0.71-1.01)	.059	0.84 (0.71-1.01)	.06
Race and ethnicity (reference group, White)						
Black	NA	NA	0.86 (0.64-1.14)	.29	0.83 (0.63-1.11)	.21
Hispanic	NA	NA	0.95 (0.52-1.72)	.85	0.92 (0.51-1.67)	.78
Other^c^	NA	NA	1.27 (0.79-2.04)	.31	1.25 (0.78-2.01)	.35
History of atrial fibrillation, yes vs no	NA	NA	0.77 (0.59-1.01)	.056	0.77 (0.59-1.01)	.06
History of stroke, yes vs no	NA	NA	0.72 (0.59-0.88)	.001	0.72 (0.59-0.87)	<.001
History of heart failure, yes vs no	NA	NA	0.92 (0.68-1.24)	.57	0.92 (0.68-1.24)	.59
History of diabetes, yes vs no	NA	NA	0.98 (0.80-1.19)	.81	0.98 (0.80-1.20)	.85
History of hypertension, yes vs no	NA	NA	0.95 (0.76-1.18)	.62	0.95 (0.76-1.19)	.65
History of cigarette smoking, yes vs no	NA	NA	1.00 (0.79-1.26)	.99	1.01 (0.80-1.27)	.95
Last known well to arrival time, per 1-min increase	NA	NA	0.99 (0.99-1.00)	<.001	0.99 (0.99-1.00)	<.001
Large vessel occlusion, yes vs no	NA	NA	1.61 (1.25-2.06)	<.001	1.57 (1.23-2.02)	<.001
NIHSS category^d^						
5-15 vs 0-4 points	NA	NA	6.51 (5.27-8.03)	<.001	6.50 (5.26-8.02)	<.001
16-20 vs 0-4 points	NA	NA	8.74 (5.39-14.15)	<.001	8.79 (5.42-14.24)	<.001
21-42 vs 0-4 points	NA	NA	6.73 (4.38-10.33)	<.001	6.68 (4.34-10.26)	<.001
Arrival method						
EMS no prenotification vs private transportation	NA	NA	0.85 (0.66-1.08)	.18	0.84 (0.65-1.07)	.16
EMS prenotification vs private transportation	NA	NA	1.06 (0.85-1.34)	.59	1.05 (0.83-1.31)	.70
Off-hours arrival, yes vs no^e^	NA	NA	0.98 (0.82-1.16)	.79	0.98 (0.82-1.17)	.82
Door-to-CT time ≤20 min, yes vs no	NA	NA	2.79 (2.28-3.42)	<.001	2.80 (2.28-3.43)	<.001
Transfer to another hospital, yes vs no	NA	NA	4.03 (1.85-8.76)	<.001	5.59 (2.34-13.38)	<.001
Metropolitan hospital, yes vs no	NA	NA	NA	NA	1.03 (0.41-2.56)	.94
Teaching hospital, yes vs no	NA	NA	NA	NA	1.36 (0.63-2.94)	.43
Neurology residency program, yes vs no	NA	NA	NA	NA	1.16 (0.66-2.05)	.61
Stroke center status						
Thrombectomy-capable vs comprehensive	NA	NA	NA	NA	1.91 (0.74-4.92)	.17
Primary vs comprehensive	NA	NA	NA	NA	0.84 (0.46-1.54)	.57
Not certified vs comprehensive	NA	NA	NA	NA	0.58 (0.07-5.20)	.62
Annual No. of acute stroke discharges						
200-600 vs <200	NA	NA	NA	NA	2.26 (0.90-5.67)	.08
>600 vs <200	NA	NA	NA	NA	2.19 (0.77-6.24)	.14

Abbreviations: AOR, adjusted odds ratio; CT, computed tomography; EMS, emergency medical services; NA, not applicable; NIHSS, National Institutes of Health Stroke Scale; OR, odds ratio.

^a^ Model 2 adjusted for patient characteristics, which included the following covariates: age, sex, race and ethnicity, medical history variables, last known well to arrival time, presence of large vessel occlusion, NIHSS, arrival method, off-hours arrival, door-to-CT time, and transfer status.

^b^ Model 3 included adjustments for the patient characteristics and hospital characteristics, including metropolitan status, teaching hospital status, presence of a neurology residency program, stroke center certification status, and annual number of acute stroke discharges.

^c^ Other race and ethnicity included Asian, American Indian or Alaska Native, Native Hawaiian or Pacific Islander, or unable to determine.

^d^ Scale of 0 to 42, with higher values indicating greater severity of stroke.

^e^ Regular (nonholiday) hours were 7:00 am to 6:00 pm, Monday through Friday.

**Table 4.** Multilevel Model of DTN Time by Telestroke Activation, With and Without Adjustment for Patient and Hospital Characteristics (n = 1673)

Coefficient	Model 1: unadjusted		Model 2: adjusted for patient characteristics^a^		Model 3: further adjusted for patient and hospital characteristics^b^	
	Estimate (95% CI)	*P* value	Estimate (95% CI)	*P* value	Estimate (95% CI)	*P* value
Initial DTN time, min	54.15 (50.17 to 58.14)	NA	77.96 (69.14 to 86.79)	NA	69.53 (52.27 to 86.80)	NA
Telestroke activation, yes vs no	9.10 (4.18 to 14.02)	<.001	7.44 (2.97 to 11.90)	.001	6.55 (2.12 to 10.97)	.003
Age, per 1-y increase	NA	NA	0.01 (−0.09 to 0.11)	.79	0.02 (−0.08 to 0.12)	.67
Sex, female vs male	NA	NA	2.84 (0.30 to 5.39)	.02	2.72 (0.18 to 5.26)	.03
Race and ethnicity (reference group, White)						
Black	NA	NA	−2.73 (−6.85 to 1.39)	.19	−2.85 (−6.93 to 1.23)	.17
Hispanic	NA	NA	5.93 (−3.06 to 14.92)	.19	5.63 (−3.33 to 14.60)	.21
Other^c^	NA	NA	−2.31 (−8.29 to 3.66)	.44	−2.14 (−8.10 to 3.82)	.48
History of atrial fibrillation, yes vs no	NA	NA	−0.51 (−4.36 to 3.34)	.79	−0.54 (−4.38 to 3.30)	.78
History of stroke, yes vs no	NA	NA	2.15 (−0.70 to 4.99)	.13	2.06 (−0.78 to 4.89)	.15
History of heart failure, yes vs no	NA	NA	3.33 (−0.93 to 7.59)	.12	3.03 (−1.22 to 7.27)	.16
History of diabetes, yes vs no	NA	NA	−1.04 (−3.90 to 1.82)	.47	−1.14 (−3.99 to 1.71)	.43
History of hypertension, yes vs no	NA	NA	−0.32 (−3.41 to 2.77)	.83	−0.50 (−3.59 to 2.58)	.74
History of cigarette smoking, yes vs no	NA	NA	−1.85 (−5.17 to 1.46)	.27	−2.32 (−5.64 to 0.99)	.16
Last known well to arrival time, per 1-min increase	NA	NA	−0.04 (−0.06 to −0.02)	.001	−0.04 (−0.07 to −0.02)	.001
Large vessel occlusion, yes vs no	NA	NA	−4.62 (−7.81 to −1.43)	.004	−4.13 (−7.33 to −0.93)	.011
NIHSS category^d^						
5-15 vs 0-4 points	NA	NA	−8.29 (−11.22 to −5.37)	<.001	−8.30 (−11.22 to −5.39)	<.001
16-20 vs 0-4 points	NA	NA	−2.16 (−7.28 to 2.97)	.40	−1.92 (−7.03 to 3.19)	.46
21-42 vs 0-4 points	NA	NA	−8.34 (−13.39 to −3.28)	.001	−8.15 (−13.19 to −3.11)	.001
Arrival method						
EMS no prenotification vs private transportation	NA	NA	−0.59 (−4.42 to 3.25)	.76	−0.33 (−4.14 to 3.48)	.86
EMS prenotification vs private transportation	NA	NA	−1.47 (−4.90 to 1.97)	.40	−1.10 (−4.52 to 2.32)	.52
Off-hours arrival, yes vs no^e^	NA	NA	2.99 (0.46 to 5.51)	.02	3.04 (0.52 to 5.56)	.017
Door-to-CT time ≤20 min, yes vs no	NA	NA	−25.67 (−29.04 to −22.31)	<.001	−25.75 (−29.10 to −22.40)	<.001
Transfer to another hospital, yes vs no	NA	NA	8.80 (1.83 to 15.78)	.013	5.18 (−2.27 to 12.63)	.17
Metropolitan hospital, yes vs no	NA	NA	NA	NA	1.84 (−7.25 to 10.93)	.69
Teaching hospital, yes vs no	NA	NA	NA	NA	10.04 (2.48 to 17.60)	.009
Neurology residency program, yes vs no	NA	NA	NA	NA	3.09 (−2.62 to 8.80)	.28
Stroke center status						
Thrombectomy-capable vs comprehensive	NA	NA	NA	NA	14.30 (5.46 to 23.13)	.001
Primary vs comprehensive	NA	NA	NA	NA	11.11 (5.12 to 17.09)	<.001
Not certified vs comprehensive	NA	NA	NA	NA	24.59 (1.52 to 47.65)	.03
Annual No. of acute stroke discharges						
200-600 vs <200	NA	NA	NA	NA	−12.38 (−23.04 to −1.73)	.02
>600 vs <200	NA	NA	NA	NA	−7.46 (−19.20 to 4.29)	.21

Abbreviations: CT, computed tomography; DTN, door-to-needle; EMS, emergency medical services; NA, not applicable; NIHSS, National Institutes of Health Stroke Scale.

^a^ Model 2 adjusted for patient characteristics, which included the following covariates: age, sex, race and ethnicity, medical history variables, last known well to arrival time, presence of large vessel occlusion, NIHSS, arrival method, off-hours arrival, door-to-CT time, and transfer status.

^b^ Model 3 included adjustments for the patient characteristics and hospital characteristics, including metropolitan status, teaching hospital status, presence of a neurology residency program, stroke center certification status, and annual number of acute stroke discharges.

^c^ Other race and ethnicity included Asian, American Indian or Alaska Native, Native Hawaiian or Pacific Islander, or unable to determine.

^d^ Scale of 0 to 42, with higher values indicating greater severity of stroke.

^e^ Regular (nonholiday) hours were 7:00 am to 6:00 pm, Monday through Friday.

**Table 5.** Multilevel Model of Guideline-Concordant Door-to-Needle Time Within 60 Minutes by Telestroke Activation, With and Without Adjustment for Patient and Hospital Characteristics (n = 1673)

Coefficient	Model 1: unadjusted		Model 2: adjusted for patient characteristics^a^		Model 3: further adjusted for patient and hospital characteristics^b^	
	OR (95% CI)	*P* value	AOR (95% CI)	*P* value	AOR (95% CI)	*P* value
Telestroke activation, yes vs no	0.52 (0.36-0.74)	<.001	0.51 (0.35-0.76)	<.001	0.56 (0.39-0.81)	.002
Age, per 1-y increase	NA	NA	1.00 (0.99-1.01)	.70	1.00 (0.99-1.01)	.91
Sex, female vs male	NA	NA	0.82 (0.64-1.05)	.11	0.83 (0.65-1.06)	.14
Race and ethnicity						
Black vs White	NA	NA	1.09 (0.74-1.61)	.65	1.10 (0.75-1.61)	.62
Hispanic vs White	NA	NA	0.56 (0.24-1.30)	.17	0.58 (0.25-1.34)	.20
Other vs White^c^	NA	NA	0.91 (0.51-1.61)	.74	0.87 (0.50-1.54)	.64
History of atrial fibrillation, yes vs no	NA	NA	0.89 (0.62-1.28)	.54	0.89 (0.62-1.28)	.53
History of stroke, yes vs no	NA	NA	0.82 (0.63-1.07)	.15	0.83 (0.64-1.08)	.16
History of heart failure, yes vs no	NA	NA	0.76 (0.51-1.14)	.18	0.79 (0.53-1.18)	.24
History of diabetes, yes vs no	NA	NA	1.18 (0.90-1.56)	.23	1.20 (0.91-1.58)	.19
History of hypertension, yes vs no	NA	NA	1.04 (0.77-1.39)	.80	1.06 (0.79-1.43)	.68
History of cigarette smoking, yes vs no	NA	NA	1.08 (0.79-1.48)	.63	1.13 (0.83-1.55)	.44
Last known well to arrival time, per 1-min increase	NA	NA	1.00 (1.00-1.01)	<.001	1.00 (1.00-1.01)	<.001
Large vessel occlusion, yes vs no	NA	NA	1.34 (0.98-1.83)	.07	1.26 (0.92-1.73)	.15
NIHSS category^d^						
5-15 vs 0-4 points	NA	NA	1.73 (1.31-2.29)	<.001	1.75 (1.33-2.31)	<.001
16-20 vs 0-4 points	NA	NA	1.33 (0.81-2.19)	.25	1.31 (0.80-2.15)	.28
21-42 vs 0-4 points	NA	NA	1.60 (0.98-2.60)	.058	1.59 (0.98-2.59)	.06
Arrival method						
EMS no prenotification vs private transportation	NA	NA	0.93 (0.66-1.31)	.67	0.89 (0.63-1.25)	.49
EMS prenotification vs private transportation	NA	NA	1.16 (0.84-1.59)	.36	1.11 (0.81-1.53)	.51
Off-hours arrival, yes vs no^e^	NA	NA	0.87 (0.69-1.11)	.26	0.87 (0.68-1.11)	.25
Door-to-CT time ≤20 min, yes vs no	NA	NA	7.26 (5.36-9.83)	<.001	7.34 (5.44-9.92)	<.001
Transfer to another hospital, yes vs no	NA	NA	0.58 (0.31-1.09)	.09	0.90 (0.45-1.77)	.75
Metropolitan hospital, yes vs no	NA	NA	NA	NA	0.91 (0.48-1.76)	.78
Teaching hospital, yes vs no	NA	NA	NA	NA	0.51 (0.30-0.88)	.02
Neurology residency program, yes vs no	NA	NA	NA	NA	0.88 (0.58-1.35)	.56
Stroke center status						
Thrombectomy-capable vs comprehensive	NA	NA	NA	NA	0.38 (0.20-0.72)	.002
Primary vs comprehensive	NA	NA	NA	NA	0.39 (0.25-0.61)	<.001
Not certified vs comprehensive	NA	NA	NA	NA	0.08 (0.01-0.59)	.01
Annual No. of acute stroke discharges						
200-600 vs <200	NA	NA	NA	NA	2.22 (0.99-4.97)	.054
>600 vs <200	NA	NA	NA	NA	1.37 (0.57-3.32)	.48

Abbreviations: AOR, adjusted odds ratio; CT, computed tomography; EMS, emergency medical services; NA, not applicable; NIHSS, National Institutes of Health Stroke Scale; OR, odds ratio.

^a^ Model 2 adjusted for patient characteristics, which included the following covariates: age, sex, race and ethnicity, medical history variables, last known well to arrival time, presence of large vessel occlusion, NIHSS, arrival method, off-hours arrival, door-to-CT time, and transfer status.

^b^ Model 3 included adjustments for the patient characteristics and hospital characteristics, including metropolitan status, teaching hospital status, presence of a neurology residency program, stroke center certification status, and annual number of acute stroke discharges.

^c^ Other race and ethnicity included Asian, American Indian or Alaska Native, Native Hawaiian or Pacific Islander, or unable to determine.

^d^ Scale of 0 to 42, with higher values indicating greater severity of stroke.

^e^ Regular (nonholiday) hours were 7:00 am to 6:00 pm, Monday through Friday.

After adjustment for patient and hospital characteristics, telestroke treatment was not associated with being discharged home, independent ambulation at discharge, or receiving DTN within 45 minutes (eTables 2-4 in Supplement 1). Among 255 patients who underwent interhospital transfer (207 patients received thrombolysis), telestroke was associated with significantly longer DIDO times (46.90 minutes longer; 95% CI, 1.08-92.72 minutes longer; *P* = .04) (eTable 5 in Supplement 1).

The sensitivity analysis that removed transferred patients produced similar results to the primary analysis (eTables 6-8 in Supplement 1). A comparison of patients with nonmissing vs missing telestroke activation status showed significant differences in several baseline and hospital-level characteristics (eTable 9 in Supplement 1), yet a sensitivity analysis that imputed missing values for telestroke activation to no yielded overall similar results to the primary analyses (eTables 10-12 in Supplement 1).

## Discussion

This cohort study of patients with AIS potentially eligible for thrombolysis found that those evaluated by telestroke were more likely to receive thrombolysis but had significantly prolonged DTN times compared with those not evaluated by telestroke. Namely, patients evaluated by telestroke had a 44% lower odds of meeting guideline-concordant DTN times within 60 minutes compared with those who were not treated by telestroke. These differences were partially attenuated by patient-level and hospital-level characteristics but remained statistically significant in fully adjusted models. Among transferred patients, telestroke was associated with significantly prolonged (47 minutes longer) DIDO transfer times.

Prior literature comparing stroke treatment times via telestroke and standard, in-person stroke care has consisted of analyses of single medical systems. A 2011 study from the University of Pittsburgh found prolonged DTN times in patients evaluated by telestroke at spoke hospitals compared with those treated at academic hub hospitals.^11^ Another analysis from 2017 found significantly prolonged DTN times for patients treated with telestroke compared with hub-treated patients and concluded that time from emergency department arrival to telestroke consultation request was the largest barrier to timely DTN times in the single telestroke network.^14^ A 2018 publication from the Mayo Clinic system found prolonged time metrics in patients treated with telestroke at spoke hospitals compared with hub hospitals, with longer times to stroke alert activation, stroke team examination, and time to treatment via telestroke.^12^ The current study substantially expands this prior work by studying 42 hospitals in the Paul Coverdell Michigan registry and controlling for patient- and hospital-level confounders.

Our finding that patients evaluated by telestroke had a 44% lower odds of achieving guideline-concordant DTN times is important because treatment for AIS is very time-sensitive,^1,3^ and delays put patients at risk for increased disability and mortality. Substantial national resources and efforts have been expended to improve DTN times. In 2010, the AHA initiated the Target: Stroke program to improve DTN times nationally and to improve stroke care and outcomes.^4^ A 2014 study evaluating Target: Stroke implementation found that hospitals participating in the initiative reduced their average DTN times from 74 to 59 minutes. Patients with stroke treated within 60 minutes had improved outcomes, including reduced long-term disability and in-hospital mortality.^6^ Given the success of Target: Stroke Phase I, the AHA and American Stroke Association commenced Target: Stroke Phase II in 2015, raising the goal for 75% of eligible patients with ischemic stroke to be treated within 60 minutes. In Target: Stroke Phase III, the bar was raised even higher, with a goal of 85% of patients being treated within 60 minutes.^7^ By contrast, our study found that from 2022 to 2023, only 60% of patients evaluated by telestroke received thrombolysis within 60 minutes. This finding represents a major opportunity for ongoing quality improvement, and improving these treatment times will require an understanding of the unique factors in telestroke systems that contribute to treatment delays.

In this study, patients evaluated by telestroke had a higher odds of receiving thrombolysis compared with those who were not. A challenge of interpreting this result is the potential selection bias that patients for whom a telestroke consultation was placed may have been inherently more likely to be candidates for acute stroke treatment, even though the same selection criteria were applied to both telestroke and nontelestroke cases. Acknowledging this potential bias, the higher thrombolytic use by telestroke in this study is consistent with a prior study using administrative claims data, which found a higher use of thrombolysis in patients receiving telestroke vs usual care (not telestroke).^8^ The strength of our study using stroke registry data was the adjustment for highly detailed patient-level (eg, stroke severity as measured by NIHSS) and hospital-level confounders (eg, Joint Commission stroke center certification status), which are not captured in claims data.

Prior literature, which has largely focused on in-person–assessed AIS cases, demonstrated that EMS prenotification is associated with faster DTN times,^21^ yet this was not the case in our study. Emergency medical service prenotification to telestroke spoke sites does not standardly alert telestroke-providing neurologists at the hub site.^14^ Thus, EMS prenotification may not yield the same time-treatment benefit for telestroke in its present form. Future studies should explore the role of EMS prenotification in telestroke systems to optimize this important systems factor.

Patients evaluated by telestroke had a significantly prolonged DIDO time (46.9 minutes longer) compared with those who were not, after accounting for clustering by hospital site and adjusting for patient-level covariates. Particularly in the era of endovascular thrombectomy, DIDO time has become an important stroke quality metric, with current recommendations for times within 120 minutes for all stroke transfers^25^ and within 90 minutes for potential candidates for endovascular thrombectomy.^26^ Prior work using the national Get With The Guidelines–Stroke registry found that DIDO times were significantly prolonged compared with these recommended targets, with an overall median DIDO time of 174 minutes for all stroke transfers.^27^ The current study found DIDO times comparable to this prior work (166 minutes for telestroke, 150 minutes for nontelestroke), but prior studies have not compared DIDO time by telestroke status. Our findings highlight that DIDO times were particularly prolonged via telestroke. A prior qualitative analysis within an academic telestroke network identified several barriers to timely transfer, including lack of EMS availability and difficulty finding an accepting facility.^28^ Future work should test these findings in other and larger samples and evaluate for additional telestroke-specific factors that may contribute to prolonged DIDO times.

In the bivariate comparisons, patients evaluated by telestroke were less likely to ambulate independently at hospital discharge and less likely to be discharged home than those who were not. The directions of the associations were maintained in the hierarchical multivariable models, but they lost statistical significance. A prior study published in 2018 from the Mayo Clinic Telestroke Program compared evidence-based stroke discharge outcomes between hub-and-spoke hospital–treated patients and found worse functional outcomes at hospital discharge among telestroke spoke–treated patients.^12^ However, this prior study only used bivariate comparisons without multivariable adjustment for patient- and hospital-level characteristics. Our findings suggest that patient and hospital characteristics contribute to the observed association between telestroke and these outcomes.

### Limitations

This study has several limitations. First, the study was based on stroke registry results from a single state. Results may differ if further examined in other states or in a national sample. Nonetheless, the Paul Coverdell Michigan Stroke Registry comprises a wide range of participating hospital types and overall accounts for approximately 64% of strokes in Michigan statewide.^29^ Second, the telestroke variable used as the primary exposure started routinely being collected in the Paul Coverdell Michigan Stroke Registry in 2022, and it had a high overall rate of missingness that may have been due to the recent introduction and less familiarity with this variable among registry abstractors. Still, the use of a telestroke-specific variable represents an important advance as prior large-scale telestroke literature has focused on telestroke capacity at the hospital level^30^ rather than on patient-level documentation of a telestroke encounter. Third, the nontelestroke comparison group in the primary analysis was a heterogeneous group of patients presenting to a variety of stroke center types (Table 1). However, with the hierarchical modeling approach, we observed that only 12% of the variation of receipt of thrombolysis and 10% of the variation in DTN time outcomes were associated with differences between hospitals. Fourth, secondary outcomes included metrics of functional ability, eg, mRS scores and ambulatory status, but these were measured at hospital discharge and not at 90-day follow-up, which is the standard follow-up time frame in many acute stroke treatment trials.^31^ Yet, discharge mRS scores have been found to accurately estimate 90-day mRS scores.^32^ Fifth, there were relatively few treated patients transferred to another hospital within the sample (which may have been due to the study’s selection criteria of eligibility for thrombolytic therapy), limiting the power of the DIDO outcome analysis. Though hospital cluster effects were accounted for via the hierarchical modeling in this study, we were unable to include hospital-level covariates in the DIDO outcome model due to limited degrees of freedom. Comparison of DIDO times by telestroke status represents an important first evaluation in this field but should be confirmed in larger datasets evaluating interhospital transfer. Finally, given the observational study design comparing different patients from different hospitals, even robust adjustment may leave residual confounding, and this study estimates associations rather than causal effects.

## Conclusions

This state registry–based cohort study of patients with AIS potentially eligible for thrombolysis found that patients evaluated by telestroke had a higher odds of receiving thrombolysis but significantly prolonged DTN and DIDO times and a lower odds of meeting guideline-concordant DTN times within 60 minutes compared with patients not evaluated by telestroke. Telestroke has the potential to revolutionize acute stroke treatment by improving access to stroke care, including increased use of thrombolysis, yet these findings suggest that there is room to improve DTN and transfer times for patients evaluated by telestroke to ensure that all patients with ischemic stroke receive guideline-concordant, time-sensitive care.

## Notes

Supplement 1. **eTable 1.** Hospital Characteristics Stratified by Telestroke Hospitals (n = 22) vs Nontelestroke Hospitals (n = 20) **eTable 2.** Multilevel Model of Discharge Destination Being Home by Telestroke Activation Among Those Who Received Thrombolysis (n = 1673) **eTable 3.** Multilevel Model of Ability to Ambulate Independently at Discharge by Telestroke Activation Among Those Who Received Thrombolysis (n = 1446) **eTable 4.** Multilevel Model of Door-to-Needle (DTN) Time (≤45 Minutes) by Telestroke Activation, With and Without Adjustment for Patient and Hospital Characteristics (n = 1672) **eTable 5.** Multilevel Model of Door-in-Door-Out time by Telestroke Activation in Participants Who Were Transferred to Another Hospital **eTable 6.** Multilevel Model of Receipt of Thrombolysis by Telestroke Activation, With and Without Adjustment for Patient and Hospital Characteristics, Excluding Participants Who Were Transferred **eTable 7.** Multilevel Model of Door-to-Needle Time by Telestroke Activation, With and Without Adjustment for Patient and Hospital Characteristics, Excluding Patients Who Were Transferred **eTable 8.** Multilevel Model of Guideline-Concordant Door-to-Needle (DTN) Time (≤60 Minutes) by Telestroke Activation, With or Without Adjustment for Patient and Hospital Characteristics, Excluding Patients Who Were Transferred **eTable 9.** Baseline Patient and Hospital Characteristics Stratified by Missingness of Telestroke Activation (n = 4259) **eTable 10.** Multilevel Model of Receipt of Thrombolysis by Telestroke Activation, With and Without Adjustment for Patient and Hospital Characteristics (Participants With Missing Telestroke Activation Are Included as No Telestroke) **eTable 11.** Multilevel Model of Door-to-Needle (DTN) Time by Telestroke Activation, With and Without Adjustment for Patient and Hospital Characteristics (Participants With Missing Telestroke Activation Are Included as No Telestroke) **eTable 12.** Multilevel Model of Guideline-Concordant Door-to-Needle (DTN) Time (≤60 Minutes) by Telestroke Activation, With and Without Adjustment for Patient and Hospital Characteristics (Participants With Missing Telestroke Activation Are Included as No Telestroke)

Supplement 2. **eFigure 1.** Study Population **eFigure 2.** Variation in Telestroke Use at the Hospital Level

Supplement 3.
Data Sharing Statement
