# Supplementary material for: Telestroke and Timely Treatment and Outcomes in Patients With Acute Ischemic Stroke
Source: JAMA Netw Open. 2025 Sep 26;8(9):e2534275. doi: 10.1001/jamanetworkopen.2025.34275 (PMC12475942; doi:10.1001/jamanetworkopen.2025.34275)
Supplement: Supplement 2. — eFigure 1. Study Population eFigure 2. Variation in Telestroke Use at the Hospital Level [file jamanetwopen-e2534275-s002.pdf]

Supplemental Online Content

Stamm B, Whitney RT, Royan R, et al. Telestroke and timely treatment and outcomes in patients with acute ischemic stroke. *JAMA Netw Open*. 2025;8(9):e2534275. doi:10.1001/jamanetworkopen.2025.34275

eFigure 1. Study Population

eFigure 2. Variation in Telestroke Use at the Hospital Level

This supplemental material has been provided by the authors to give readers additional information about their work.

© 2025 Stamm B et al. *JAMA Network Open*.

eFigure 1. Study Population

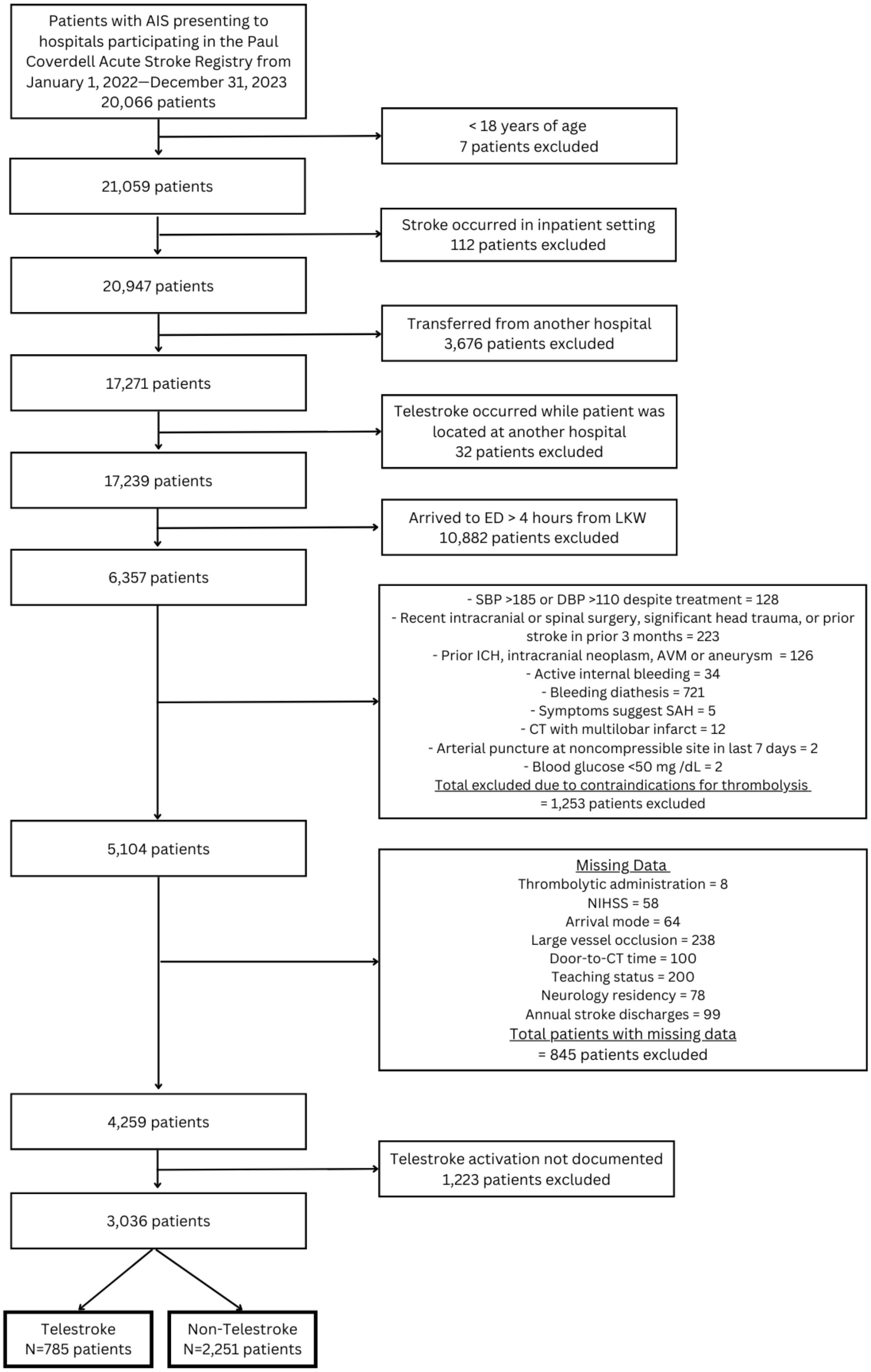

AIS: Acute ischemic stroke; ED: Emergency Department; SBP: Systolic blood pressure; DBP: Diastolic blood pressure; LKW: Last known well; SAH: Subarachnoid hemorrhage; AVM: Arterial venous malformation; NIHSS: National Institute of Health Stroke Scale

© 2025 Stamm B et al. *JAMA Network Open*.

eFigure 2. Variation in Telestroke Use at the Hospital Level

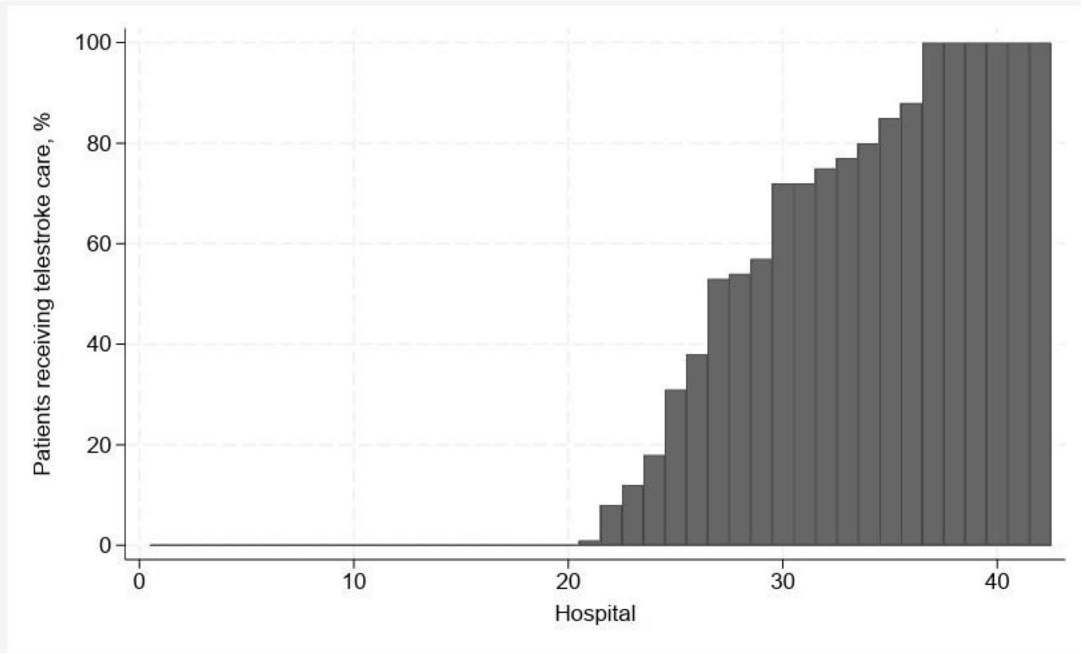

© 2025 Stamm B et al. *JAMA Network Open*.
