# Supplementary material for: Telestroke and Timely Treatment and Outcomes in Patients With Acute Ischemic Stroke
Source: JAMA Netw Open. 2025 Sep 26;8(9):e2534275. doi: 10.1001/jamanetworkopen.2025.34275 (PMC12475942; doi:10.1001/jamanetworkopen.2025.34275)
Supplement: Supplement 3. — Data Sharing Statement [file jamanetwopen-e2534275-s003.pdf]

Data Sharing Statement

Stamm. Telestroke and Timely Treatment and Outcomes in Patients With Acute Ischemic Stroke. *JAMA Netw Open*. Published September 26, 2025.
doi:10.1001/jamanetworkopen.2025.34275

Data
Data available: No

Additional Information
Explanation for why data not available: The data used in this study are derived from the Paul Coverdell Michigan Stroke registry, which is overseen by the Michigan Department of Health and Human Services. Due to the sensitive nature of the data, this cannot be broadly shared, but individual data access requests can be made through this organization.
